# Equalising power imbalances or a trail of broken promises? A qualitative study on engaging people with diverse lived experience of marginalisation in food policymaking in Australia

**DOI:** 10.1186/s12889-025-21733-4

**Published:** 2025-02-14

**Authors:** Carolina Venegas Hargous, Kevin Kapeke, Kathryn Backholer, Dheepa Jeyapalan, Veronica Nunez, Jennifer Browne, Anna Peeters, Alexandra Chung, Steven Allender, Victoria Stead, Yin Paradies, Christina Zorbas

**Affiliations:** 1 Global Centre for Preventive Health and Nutrition, School of Health and Social Development, Institute for Health Transformation, Faculty of Health, Deakin University, 1 Gheringhap Street, Geelong, 3220 Australia; 2 The Victorian Health Promotion Foundation (VicHealth), Level 2, 355 Spencer Street, West Melbourne, Melbourne, 3003 Australia; 3 Department of Nutrition, Dietetics and Food, Monash University, Level 1, 264 Ferntree Gully Road, Notting Hill, Melbourne, 3168 Australia; 4 School of Humanities and Social Sciences, Faculty of Arts and Education, Deakin University, 1 Gheringhap Street, Geelong, 3220 Australia; 5 Alfred Deakin Institute, Faculty of Arts and Education, Deakin University, 1 Gheringhap Street, Geelong, 3220 Australia

**Keywords:** Lived experience, Marginalised voices, Systematic exclusion, Meaningful engagement, Food policy, Policymaking, Decision-making

## Abstract

**Background:**

Achieving nutrition and health equity warrants understanding lived experiences of marginalisation. Yet, people with diverse lived experiences are often inadequately included in food policy advocacy, agenda setting, and development. We aimed to explore cross-sectoral perceptions of engaging people with lived experiences of marginalisation in food policymaking in Australia, specifically in terms of challenges, enablers, required actions, and potential outcomes of doing so.

**Methods:**

In-depth semi-structured interviews were conducted with 24 people with expertise in food policy and/or community engagement from academic, government, advocacy, and community sectors. Interviews were inductively and deductively coded using the Knowledge-to-Action framework.

**Results:**

Participants identified few food policymaking examples where people with lived experience have been meaningfully engaged. Reported barriers included the lack of time, resources, and prioritisation across sectors and the lack of political commitment to inclusive policymaking. Having access to successful examples, existing networks of actors and flexible funding were among the few enablers identified. Several actions were deemed necessary to effectively engage people with lived experience in food policymaking and improve current practice: (1) having a dedicated budget; (2) enabling true collaboration where people with lived experience are valued, effectively engaged, sufficiently represented, have the opportunity to work alongside decision-makers, and where power is equalised; (3) striving to do no harm to the people engaged; and (4) ensuring results from engaging people with lived experience are effectively disseminated.

**Conclusions:**

We provide a list of practical recommendations to guide more inclusive, equitable and fit-for-purpose food policymaking into the future. These recommendations seek to challenge dominant systems of discrimination by demonstrating how we can tangibly shift to ways of working that value and elevate the power of people who are often excluded from many decision-making systems, specifically when it comes to food and nutrition.

**Supplementary Information:**

The online version contains supplementary material available at 10.1186/s12889-025-21733-4.

## Introduction

Populations around the world are experiencing high burdens of food insecurity, malnutrition, and diet-related non-communicable diseases (NCDs), such as cardiovascular diseases, cancers, and type 2 diabetes [1]. Adverse nutrition and diet-related health outcomes disproportionately affect people experiencing the greatest social and/or economic disadvantage and intersectional forms of marginalisation (hereafter people with lived experience of marginalisation), including in high-income countries like Australia [2, 3]. For example, previous research shows that people with lived experience of socioeconomic marginalisation in Australia (i.e., people from low-income households receiving government income support) face multiple barriers to prioritising healthy eating, including competing financial priorities such as housing and education, with food being one of the most flexible parts of their budgets [4]. Moreover, physical barriers such as housing location determine proximity to fresh food stores and transportation, often posing additional barriers to accessing healthy food options [4]. Despite these evident challenges, people with lived experience of marginalisation in Australia often express a dominant perception that public and policy rhetoric misrepresents their entrenched struggles of being able to access and afford high-quality food in a dignified manner [4].

Government and non-government actors recognise that equity-oriented policies are urgently needed across sectors to effectively reduce the extensive health and social impacts of diet-related issues [5–8]. Equity-oriented public health policy creates fairer conditions that enable everyone to achieve their full health potential, including by addressing imbalances in the distribution of power, money, and resources across society [8, 9]. For food and nutrition, this can include creating enabling food environments that support healthy and affordable diets, for all, through nutrition-specific (e.g., healthy food subsidies) and nutrition-sensitive (e.g., social protection) policies [10]. Research shows that whilst governments often acknowledge the need to act on the upstream determinants of health and nutrition, seldom does this translate into action [8, 11–13].

As Table 1 outlines, the prioritisation of equity in food and nutrition policy agendas (herein abbreviated to ‘food policy’) is frequently overlooked, with inadequate acknowledgment of systems of exclusion, unfairness and injustice (e.g., neoliberalism, racism, paternalism, classism, ablism, etc.) that continue to dominate policy processes, including by prioritising the interests of a few powerful individuals or groups [14]. According to the nutrition equity framework [15], these mainstreamed systems of exclusion result in social stratification and the worsening of diet-related health inequalities [2, 3]. Other evidence collectively indicates that dominant sociopolitical systems facilitate the prioritisation of commercial over public health interests [15], insufficient attempts to include community voices and experiences in policy decision-making [15], the framing of health as an individual responsibility and people who experience marginalisation as irresponsible [14], and the adoption of behaviour change strategies instead of actions on the social determinants of population diets [14].

**Table 1:** A comparison of status quo and equity-oriented food, nutrition and health policymaking

Nutrition/health policymaking	Status quo	Equity-oriented
Ideologies	• Neoliberalism • Racism and mono-culturalism • Paternalism • Biomedical	• Social democracies • Human Rights • Self-determination
Policy actors	• Siloed governments • High-level government officials • Commercial sector interference • Biomedical community	• Whole-of-government approach • Intersectoral policy networks • Civil society mobilisation: priority populations, general public, non-government organisations (NGOs), opinion leaders, Aboriginal community-controlled organisations
Problem framing/ dominant discourses	• Individualisation of health outcomes • Priority populations portrayed as problematic (i.e., othered, irresponsible, incompetent, deficit discourse)	• Emphasis on social and structural determinants of health outcomes • Evidence-based • Strengths-based
Evidence	• Not essential • Selective use of evidence	• Policy priorities determined by evidence on health inequalities • Use evidence of lived experience and acceptability of policy options
Public participation	• Nonparticipation (e.g., manipulation, therapy/education) and tokenism (e.g., informing, consulting and placation)	• Public control through partnerships, delegated power and public-controlled decision making
Policy outcomes	• Individual behaviour change strategies prioritised • Biomedical approaches prioritised over public health • No change to the status quo (i.e., no reduction in health inequalities)	• Structural actions on the social determinants of health prioritised across government levels and sectors • Citizen empowerment • Effective, fit-for-purpose policies designed, implemented and evaluated for equity impacts

Table informed by:

Baker P, Friel S, Kay A, Baum F, Strazdins L, Mackean T. What Enables and Constrains the Inclusion of the Social Determinants of Health Inequities in Government Policy Agendas? A Narrative Review. International Journal of Health Policy and Management. 2018;7(2):101 − 11

Arnstein SR. A Ladder Of Citizen Participation. Journal of the American Institute of Planners. 1969;35(4):216 − 24

Zorbas C, Browne J, Chung A, Baker P, Palermo C, Reeve E, et al. National nutrition policy in high-income countries: is health equity on the agenda? Nutrition Reviews. 2020

Strengthening the inclusion of diverse voices and experiences, whilst challenging current exclusionary decision-making systems, can help shift current food policy to align with health equity goals [15, 16]. For example, evidence has demonstrated that incorporating the voices of Aboriginal and Torres Strait Islander peoples in Australian policy discourse advances food policies that are more inclusive, culturally safe, and aligned with self-determined First Nations priorities [17, 18]. Foregrounding values, voices, and lived experiences of people harmed by marginalisation in food policymaking also aligns with approaches like deliberative democracy [19], Human Rights, critical theory [20], ethnography [20], the Advocacy Coalition Framework [21], and citizen science [22, 23]. Nevertheless, internationally, few attempts have been made to meaningfully engage people with diverse lived experiences in food policymaking beyond community consultation [15]. Arnstein’s renown Ladder of Citizen Participation argues that these forms of consultation are often limited because they risk being symbolic gestures of goodwill (i.e., tokenistic) that have limited impacts on decision-making processes [22]. While some international literature on how to effectively engage people with lived experience of marginalisation in policy and decision-making processes exists in other health sectors [24–26], it remains unclear how people with lived experience of marginalisation can be best engaged and meaningfully participate in food policymaking, particularly in the Australian context. To fill this gap, we aimed to explore cross-sectoral perceptions of engaging people with lived experience of marginalisation in food policymaking, predominantly as it relates to Australia, in terms of challenges, enablers, required actions, and potential outcomes of doing so. Findings from this study will inform practical recommendations for fostering inclusive and equitable food policymaking, that is fit-for-purpose and can be applied across sectors.

## Materials and methods

A qualitative study was conducted using in-depth semi-structured interviews. The COnsolidated criteria for REporting Qualitative research (COREQ) guidelines [27] were used as reporting guidelines.

### Researcher positionality

Members of the research team currently work in the fields of public health, nutrition, equity, politics, and/or anthropology and have experience conducting qualitative research. As academics and practitioners (KK, DJ, VN), we occupy positions of socio-economic and cultural privilege, including high levels of literacy in relation to nutrition and food policy. Different members of the team also have relevant lived experience related to colonial and gendered marginalisation, as well as experience working with communities who experience health inequity in Australia and globally. Our research focuses on supporting programs and policies to promote equity by addressing the systemic determinants of health and social inequalities. This action-oriented research project was thus informed by our common interests in exploring initiatives to strengthen the inclusion of diverse peoples in public policy. The research was funded by the Victorian Health Promotion Foundation (VicHealth) and aligned with the organisation’s strategic priorities to promote policies that support diet-related health equity.

### Theoretical framework

This research draws on critical theory; seeking to critique how current social structures drive power imbalances in decision-making and diverse experiences of diet-related health inequalities [28]. We utilised the Advocacy Coalition Framework [21] to understand how advocates with similar beliefs can influence policies over time and the Knowledge-to-Action implementation science framework to ensure our research focused on identifying practical policy recommendations [29]. Particularly, the Knowledge-to-Action framework firstly comprises a knowledge creation cycle, which focuses on generating and translating knowledge into usable formats. The second action cycle focuses on applying this knowledge through a seven-step process: problem identification (i.e. why should lived experience of marginalisation be included in food policy?), knowledge adaptation (i.e. how can lived experience be included in current practice?), assessment of barriers to knowledge use, selection of information (e.g. identification of best practices), tailoring and implementation of interventions, monitoring knowledge use, outcome evaluation, and sustaining the use of knowledge [29]. These frameworks and the available literature summarised in Table 1 guided participant recruitment, interview schedules, and data analysis.

### Participant selection

Representatives from academia, NGOs, government, advocacy, and community organisations were eligible to participate in our study if they had expertise in food or social policy and/or community engagement. Purposive sampling was used to identify key actors and advocates from well-known health and social policy bodies in Victoria, where this research was conducted, and more broadly across Australia and internationally, where well-known initiatives to engage community lived experience were identified. In particular, actors with global perspectives were recruited where the research team identified strong expertise likely to enhance our understanding of the topic. Recruitment of some actors was also prioritised due to them identifying with culturally diverse, Aboriginal, regional and homeless lived experiences (although such identifying information was not collected to protect participant confidentiality). Snowball sampling was used by asking participants to identify other potentially relevant interviewees. Whilst a sub-sample of interviewees (predominantly from the academic sector) were known to the research team, the lead researcher did not have close personal connections with any participant. We sought to recruit relatively equal representation across the sectors listed above. Approximately 40 people were approached via email, with 24 agreeing to participate (in 21 interviews). Reasons for non-responses were not provided.

### Setting

Interviews were conducted online via Zoom where only participants and researchers were present. All interviewees were based in Australia, except for two participants with global perspectives who were based in the UK and South Africa.

### Data collection

Interviews were guided by an original semi-structured interview guide (not previously published) containing 13 open-ended questions (see Table S1 in Supplementary Material) that were based on the aforementioned theoretical underpinnings [21, 29]. In general, question topics related to understanding participants’ perceptions of the current status quo of inequities in population nutrition, and how the voices, values, and lived experiences of people experiencing marginalisation can contribute to more equitable food policymaking. Prompts were provided to encourage participant responses where necessary. Interviews typically lasted 45–60 min. The second and senior authors conducted the interviews, with audio recordings obtained using video conferencing software (Zoom).

Once information power was obtained, the sample was deemed complete. We defined information power as the point at which the different interviewee groups were balanced in their representation and had provided in-depth dialogue on the topic that could inform a comprehensive analysis [30]. Audio recordings were transcribed by Zoom or SmartDocs and interview transcripts were returned to participants for member-checking and editing. Four participants returned edited transcripts, with edited versions used in the analysis alongside all other unedited transcripts.

### Data analysis

Two authors (CVH and CZ) inductively coded two interviews using NVivo 12. Both authors independently developed a coding framework and initial major and minor themes that aligned with constructs of the Knowledge-to-Action framework. Interview data were most aligned with constructs related to identifying: the barriers for engaging people with lived experience of marginalisation in food policymaking (assessment of barriers to knowledge use), best practice, enablers and adaptations to current practice to better include diverse lived experiences in current food policy processes (knowledge adaptation, selection, tailoring and implementation of interventions), and outcomes of effectively engaging people with lived experience of marginalisation in food policymaking (outcome evaluation) [29]. The first author proceeded to deductively code the remaining dataset according to the coding framework. Major and minor themes describe participants’ perspectives of the barriers, enablers, practical actions, and potential outcomes of meaningfully and ethically engaging people with lived experience of marginalisation in food policymaking. Examples of initiatives where people with lived experience of marginalisation have been included in other health and social policy processes were also described. Quotes have been used to illustrate each minor theme.

### Ethics

Ethics approval for this study was obtained by the Deakin University Human Research Ethics Committee (2022_216). Written informed consent was obtained from all participants. Community representatives who were recruited to participate in capacities outside of their professional roles (*n* = 4) were reimbursed with $AU 50 for their time.

## Results

### Sample characteristics

Twenty-four participants were interviewed (83% female), including five researchers, nine community engagement experts or advocates, and ten local and State government employees. Table S2 outlines the types of organisations and roles participants were engaged in, using high-level descriptions to protect participant identities.

### Thematic overview

Findings revealed a paucity of examples that participants could identify where people with lived experience of marginalisation had been engaged to advance equitable food policies. Two participants identified examples of community consultation where local governments or local organisations have engaged people with lived experience of marginalisation to discuss barriers to healthy eating to inform local policies and initiatives. Beyond food policy, two participants identified ‘100 families WA’ as a successful example of community engagement where people experiencing marginalisation were involved as partners in a collaborative research project to discuss the issue of socioeconomic disadvantage, including problems around housing and food insecurity, informing public sector efforts and advocate for further policy action. Another participant representing the government mentioned the existence of a ministerial advisory committee formed by a diverse group of people with lived experience of marginalisation who advised the ministry on the design and implementation of a range of initiatives, from mental health recommendations to climate action policies. Similarly, two participants from NGOs identified examples where organisations have established research advisory committees and engagement committees to enable community participation from the design through to the implementation of youth-related projects, often including policy advocacy.

Reflecting on the lack of examples in the food policy space, participants identified multiple overlapping barriers to engaging people with lived experience of marginalisation in food policymaking and fewer enablers (summarised in four and two minor themes, respectively). Several actions were also discussed as necessary to improve current practice and enable true collaboration that went beyond tokenistic forms of community consultation (summarised in four minor themes). Lastly, participants described perceived outcomes of meaningfully engaging people with lived experience of marginalisation in food policymaking (summarised in four minor themes).

**Fig. 1:**
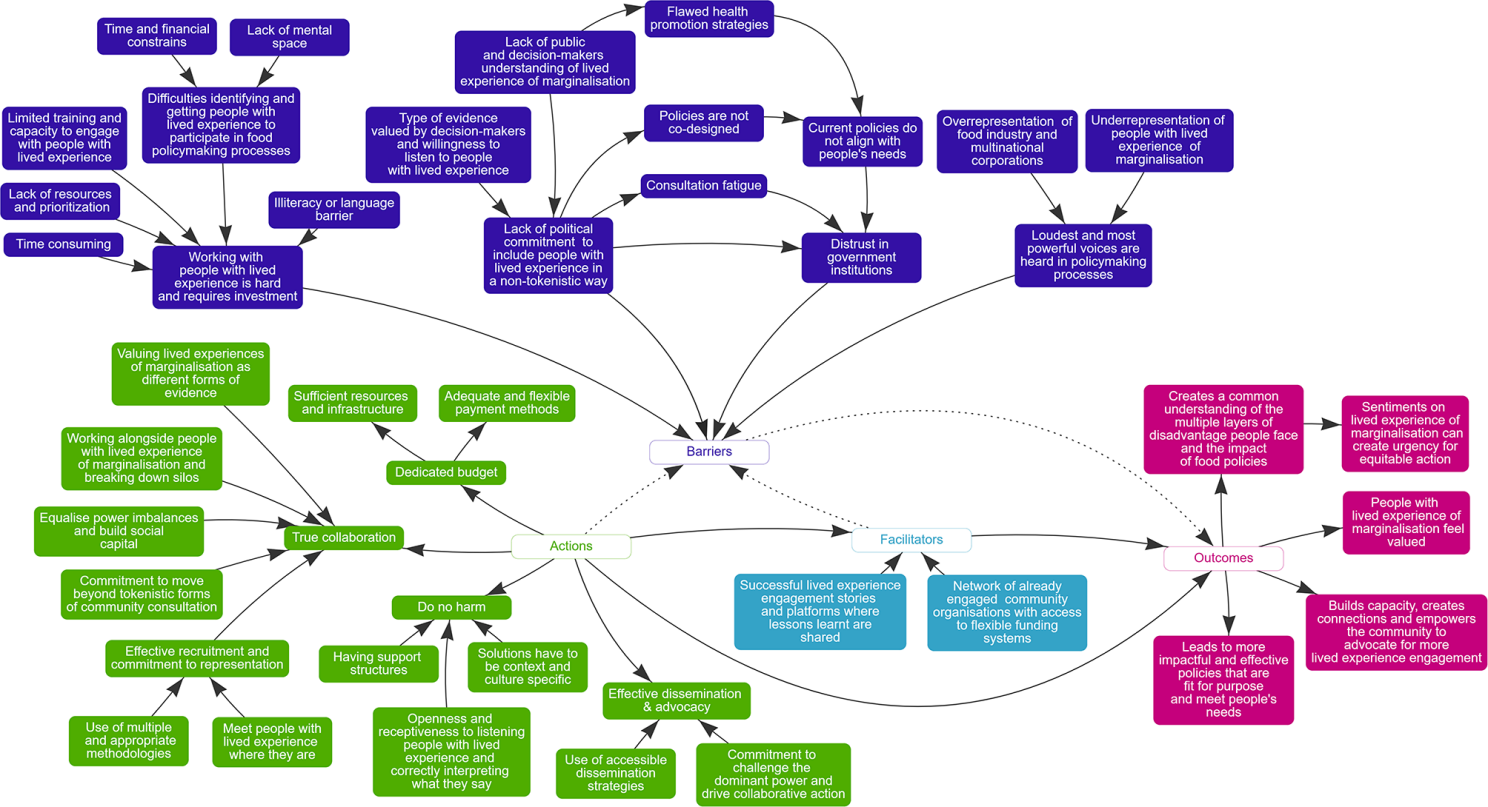
Mind map summarising participant’s knowledge on barriers and enablers to, actions required for, and outcomes of engaging people with lived experience of marginalisation in food and nutrition policymaking. Solid arrows represent direct relationships between factors, and dotted arrows represent indirect relationships between factors

These themes were collectively summarised in a mind map (see Fig. 1).

#### Barriers to engaging people with lived experience of marginalisation in food policymaking

Participants identified several interconnected barriers to engaging people with lived experience of marginalisation in food policymaking, from policy advocacy to policy development.

1. Working with people with lived experience of marginalisation requires investment in human and financial resources.

Most participants agreed that working with people with lived experience of marginalisation is time-intensive and requires the allocation and prioritisation of resources, which are often limited in research and policymaking. The lack of training and capacity-building opportunities about how to engage people with lived experience of marginalisation was identified as another barrier to conducting this work. As one participant said:

*“I think it’s very different, a different approach, so it takes a lot of time. As I said, to sort of build those relationships and build that trust, which is really, really important, it takes a very different skill set, I would say, to designing questionnaires… it takes, yeah, a different way of sort of viewing the world I think and how you value other people’s time.” (Participant 1)*.

Some participants also mentioned that identifying a representative group of people with lived experience of marginalisation can be difficult. Moreover, getting them to engage and participate in food policymaking was perceived to be an even greater challenge. One participant reflected on how:

*“I tried my best to ensure that there was diversity within the group, but I found it really difficult. I tried to ensure that there were men and women in the group, that we had LGBTIQA + representation, First Nations, young people and different neurodiversity. But it was quite difficult, and we’ve ended up with mostly women, mostly White. Our youngest participant is I think 24. It’s very important but easier said than done.” (Participant 2)*.

Difficulties engaging people with lived experience of marginalisation in food policymaking were thought to be associated with community members’ time and financial constraints, as well as competing mental and emotional priorities. Additionally, as highlighted by one participant, language and literacy barriers represented another possible challenge for engagement, particularly when working with groups of migrants and refugees.

2. Lack of political commitment to including people with lived experience of marginalisation in food policymaking.

The perceived lack of political and broader commitment to including people with lived experience of marginalisation in food policymaking was identified as another major barrier. Participants suggested that this may reflect the lack of public and decision-makers’ understanding of diverse lived experiences and the lack of importance given to including such voices in food policymaking. This was thought to translate into an unwillingness to listen and consider peoples’ lived experiences as a valuable source of knowledge and evidence. As one participant said:

*“… policymakers would rather pay a consultant $120*,*000 to do a very quick, easy piece where they ask exactly the same questions over and over again… They keep getting the same answers that they want… They’re not actually looking for the truth, so I think that’s where the problem is.” (Participant 3)*.

Participants perceived that limited public and political understanding of diverse lived experiences of marginalisation was linked to the design and implementation of flawed health promotion strategies that may be more harmful than beneficial to people experiencing marginalisation, which often devalue their life circumstances. Specifically, participants critiqued population food and nutrition policies that are not co-designed and do not meet the needs of people experiencing marginalisation. For example:

*“What’s labelling going to do for someone who can’t afford to eat?…the determinants of food insecurity is income… it’s financial. It’s not so much about food itself. Food is almost the Band-Aid approach to it. You know about food banks. It’s just a stop gap for a big structural issue.” (Participant 4)*.

3. Distrust in government institutions.

Multiple participants discussed tokenistic forms of community engagement as failures to meaningfully commit or follow through to actions that reflect people’s lived experiences and asks, leading to consultation fatigue and distrust in government institutions. This was thought to decrease the willingness of people with lived experience of marginalisation to engage and participate in food policymaking. As one participant said:

“*What’s in it for them? Why would they (engage)? Even with a $50 voucher? Why would they take part in a conversation like that? In the weight space, I’ve had people say to me “is it going to result in any real change? Because I’m not doing it if it’s not.”*” *(Participant 5)*.

Reflecting on Aboriginal and Torres Strait Islander communities’ policy consultation fatigue, one participant said:

*“One of the things that comes up a lot in relation to Aboriginal communities and consultation processes is people express a great deal of frustration about being consulted multiple times with the same issue, with the same or worsening outcomes and the information is, oh bizarre, here’s the lovely new strategy, here’s a wonderful Royal Commission with fabulous recommendations. Oh, yeah great, and they are great, and they never get implemented and no funding is put forward to enable the implementation for the strategies… if you break faith with any group or any person with whom faith has been broken on multiple different occasions you’re never going to make a connection with them again… There are some people who have had the rough end of the broken promise trail more than most. And this is a community that’s had many, many broken promises. You know, 240 years.” (Participant 6)*.

Establishing and/or recovering trust in government institutions was, hence, viewed by some participants as one of the biggest challenges towards inclusive food policymaking.

4. Opportunities to be heard are given to the most powerful and loudest voices

Most participants agreed that a key barrier to engaging people with lived experience of marginalisation was that the most powerful voices, such as those from the food industry and large multinational corporations, are usually overrepresented in food policymaking. In contrast, the voices of people with lived experience of marginalisation, including people living with disability, youth, single mothers, people experiencing homelessness, Aboriginal and Torres Strait Islander people, migrants, refugees, LGBTQ + communities, among others, were thought to be typically unheard. Reflecting on their own experience of feeling excluded from policymaking as an Aboriginal person and how hard it is for people to challenge systems of exclusion, one participant expressed that:

*“…whenever I’ve tried to get invited to these meetings with the policymakers to talk about Aboriginal nutrition, I’m never allowed through the door. I’m never invited to anything. I’m a PhD, I’m Aboriginal, I’ve got all the research backgrounds, but I’m still not good enough to be allowed to talk about Aboriginal nutrition policy. Whereas the people in the room are usually bureaucrats… They make decisions on behalf of their Ministers. And then all the other people in the room are non-Aboriginal dietitians or nutritionists. So I’m never allowed in that room. So in terms of implementation failure, I think if I can’t get in a room then, to have a voice in this process, what hope has anybody else got, really?” (Participant 7)*.

#### Enablers to including people with lived experience of marginalisation in food policymaking

Compared to the barriers, which were extensively discussed, few participants mentioned enablers for effectively engaging people with lived experience of marginalisation in food policymaking more broadly. Enablers included:

1. Having a network of already engaged community organisations with access to flexible and community-led funding systems.

Two participants mentioned that having an existing network of already engaged community organisations with access to flexible funding systems can facilitate the identification of key actors and the implementation of targeted initiatives. One participant commented on the importance of this enabler in the context of public health initiatives in a UK town:

*“I think [the town] has got a long history of community development and advocacy, I guess, so there are lots and lots and lots of engaged community organisations. So there’s sort of a pulse on what people are experiencing, knowing who’s vulnerable and where… Also… I think we’ve got like fairly flexible funding systems for these community groups, so there’s a part of funding there, but then exactly how it’s used can be more designed by the community, so I think that really helps. And it might be that the community wants something very different to what you think would be good. Might want a salsa class and that’s good too.” (Participant 1)*.

2. Having successful examples of community engagement and platforms where lessons learned are shared.

The same two interviewees additionally mentioned that having examples where people with lived experience of marginalisation have been meaningfully engaged and platforms where success stories are shared can help promote the uptake of inclusive food policymaking:

*“…what they try to do is combine their brain power and lessons learned and experiences of what’s been really helpful and share it and I think that’s really, really useful.” (Participant 1)*.

#### Actions to enable the inclusion of people with lived experience of marginalisation in food policy, research, and practice

After identifying barriers and enablers for meaningful community engagement, participants discussed actions required to make food policy, research, and practice more inclusive for people with lived experience of marginalisation. These included:

1. **Have a dedicated budget**.

Participants agreed that a key action required for achieving meaningful engagement of people with lived experience of marginalisation was having a dedicated budget that provides not only the resources and infrastructure to effectively do so but also a budget that ensures adequate and flexible payment methods to reimburse participants for their time. As one participant said:

*“…you need to make sure, as I said, you’ve got all the right infrastructure in place to be able to do it effectively. So having budget… having the infrastructure in place and making sure you’ve got budget to be able to remunerate those people… yeah, when you’re ready and all those other things are in place and you know you’ve got support around you if it’s you that’s going to be supporting the group, then the recruitment needs to be done effectively. So, I would partner again with someone with lived experience to help, who is already, you know, in a higher role to help you set up that recruitment process” (Participant 8)*.

Three participants also mentioned the importance of considering potential tensions around payments where people might agree to share their story only for financial reward.

2. **Enable true collaboration**.

Another critical action identified by most participants was the need to enable true collaboration. True collaboration was perceived as a meaningful engagement process where different forms of evidence are valued, people with lived experience of marginalisation are given opportunities to work alongside researchers and decision-makers throughout all stages of the process (i.e., agenda setting, policy formulation, policy adoption, implementation and evaluation [31]), and where all collaborators stop thinking and working in silos. As one participant said:

*“If you really want to make a difference, you have to work alongside the group you’re making the difference for” (Participant 8)*.

Participants further emphasised that true collaboration needs to equalise power imbalances and build social capital so that the power of people with lived experience of marginalisation is amplified enough for them to lead actions:

*“Empowerment is one of those key things that you need to be considering. We want to hand power over to these people. There’s historically been a huge imbalance.” (Participant 2)*.

Also, several participants agreed that true collaboration needs to be underpinned by a genuine commitment to move beyond tokenistic forms of community consultation. Tokenistic consultation was perceived as the dominant form of symbolic consultation, where actions are insufficiently implemented in response to community experiences and communities are not given the power to be involved in decisions about these actions. For example, one interview suggested:

*“… don’t be an ask hole… we’ve been a bit of an ask hole in that we’ve asked you and then we’re not doing anything with the feedback that we’ve asked for” (Participant 5)*.

Lastly, participants reflected on the importance of committing to diverse representation and ensuring people with lived experience of marginalisation are effectively recruited and engaged using flexible and appropriate methods that work for them. One participant described that:

*“… if you’re going to be genuine in your commitment to including these people, then you’ve got to be willing to work with them and work around what works for them” (Participant 9)*.

3. **Do no harm**.

Another key action highlighted by most participants was to strive to do no harm when engaging people with lived experience of marginalisation in food policymaking. Specifically, participants stressed the need for having support structures that prevent and manage potential distress (e.g., having a support person, having debrief sessions, scheduling follow-up calls) and ensuring researchers and decision-makers are willing to listen, receptive to hearing, and correctly interpret people’s stories. As one participant said:

*“…bad co-design hurts people… especially if it’s not trauma informed. You can really open wounds in people who are then not supported, who then go into crisis. I see this happen all the time…” (Participant 10)*.

Another important consideration in avoiding harm through engaging people with lived experience of marginalisation was to ensure the implementation of context and culture-specific solutions. As one participant said:

*“We know that one-size-fits-all, it’s never going to be a one-size-fits-all solution. I think place-based is best…” (Participant 11)*.

4. **Ensure effective dissemination and advocacy**.

The final action highlighted by most interviewees was to ensure results from engaging people with lived experience of marginalisation are effectively disseminated and used for policy advocacy purposes. This was thought to entail different participatory approaches and communication strategies, including photovoice, mapping, storytelling videos, vignettes, social media posts, infographics, among others, to challenge dominant power imbalances and drive collaborative policy action. For example, one participant said:

*“It was told in a way that it was very simple, it wasn’t complicated. And then the solutions are simple as well… It’s almost how do you create that narrative without misrepresenting the story? And how do you do it in a way that a decision-maker, or whoever’s reading it or taking it into account can see, “Oh, that’s a barrier, I can remove that, or that’s a need, I can fill that.”… So, I think it’s not just about the person telling the story, it’s about how you tell their story in a way that is going to get change. And having those little, kind of, vignettes of the spoken word…” (Participant 12)*.

#### Outcomes of effectively including people with lived experience of marginalisation in food policymaking

All participants reflected on the importance of including people experiencing marginalisation in food policymaking and identified several benefits of doing so.

1. **Creating a common understanding of the multiple layers of disadvantage people face**.

Participants identified that a key outcome of including people with lived experience of marginalisation in food policy, research, and practice is that it can unpack multiple layers of disadvantage and create a common understanding of the issues people face. According to one participant:

*“… there’s something about the lived experience research that has the power to, I think, generate a common understanding, a common narrative of the issue and to extend the experience of food and security and malnutrition beyond the person and they’re starting to see it as more of a systemic issue.” (Participant 13)*.

Participants agreed that having a common understanding of lived experiences of marginalisation can evoke sentiments of disappointment, sadness, compassion, and anger towards the system and can create urgency for equitable food policies.

2. **Enabling people with lived experience of marginalisation to feel valued**.

Participants also mentioned that genuinely including and valuing people with lived experience of marginalisation in food policymaking can support them to feel dignified. As one participant said:

“*I think it helps with dignity, and feeling valued and included, and having your voice heard on something that might otherwise, I don’t know, not be considered important amongst all of the other life priorities, and say policy priorities for a government, if this were to be designated as one. I think people feel valued and like to be heard on this issue.” (Participant 14)*.

3. **Building capacity, creating connections, and elevating community power**.

Furthermore, participants thought that meaningful engagement of people with lived experience of marginalisation in food policymaking can build community capacity and confidence to tackle challenges. Mechanisms underlying capacity building included creating new connections and amplifying the power of community to drive change and advocate for more engagement of people with lived experience of marginalisation in research and practice. For example, in one participant’s experience:

*“when I’ve seen lived experience research done really well, there can be an empowerment aspect or it can lead to a process of empowerment where people find that… sharing their experience somehow includes them in a process that they were excluded from before and that can spark or be an opportunity to do more of that, and maybe advocate more in the community, and build ties and connections to universities (and) decision-making processes that they might have been excluded from before” (Participant 1)*.

4. **Designing more impactful and effective food policies**.

Finally, participants frequently alluded to how people with lived experience of marginalisation know exactly what they need. As such, including them in food policymaking can inform the design of more impactful and equitable food policies that are fit-for-purpose, align with people’s priorities, and avoid potential unintended consequences. This can be summarised through one participant’s statement that:

*“You would do a better job. You will avoid a lot of pitfalls if you are creating and shaping policy and programs. You’ll get a much better product at the end… And you will get a product that won’t widen inequality, that hopefully will reduce inequality, and that would be a really good thing… So yeah… I think you’ll get a better outcome; there’s no doubt about it.” (Participant 12)*.

## Discussion

This research combines insights from community, government and research representatives on the barriers and enablers for including people with lived experience of marginalisation across all stages of food policymaking and the key actions required to improve current practice. Participants identified few examples of where people with lived experience of marginalisation had been optimally engaged in food policymaking (from research and advocacy to policy development) in a predominantly Australian context. Multiple interconnected barriers to doing so were identified, including: (i) the lack of time, resources, and prioritisation across sectors, (ii) the lack of political commitment to include diverse voices of lived experience of marginalisation (which might be partially explained by the slow nature of government processes and the high turnover rate within governmental positions), (iii) the subsequent distrust in government institutions from the people who have not been meaningfully engaged, and the (iv) overrepresentation of powerful voices in policymaking, such as large food corporations. These barriers outweighed the number of enablers participants identified for meaningfully engaging people with lived experience of marginalisation in food policymaking, which included having access to an existing network of community organisations with flexible and community-led funding systems and successful examples to learn from.

Using the Knowledge-to-Action framework, we identified several actions that participants thought could overcome barriers, enhance enablers, and ultimately achieve more inclusive food policy, research, and practice (including advocacy) in Australia. These included having a dedicated budget; enabling true collaboration by valuing and elevating the power of people with lived experience of marginalisation; striving to do no harm while engaging people with lived experience; and effectively disseminating community experiences and policy asks through inclusive food policy advocacy.

The vast majority of participants agreed that if policymakers were to act on the priorities of people with lived experience of marginalisation, there would be a wide range of positive outcomes. Indeed, there was consensus that meaningful engagement of people with lived experience of marginalisation in food policymaking enables a common understanding of the multiple layers of disadvantage people face and the impact of food policies in their lives; supports people with lived experience to feel valued and included, and hence dignified; builds their capacity to participate in food policymaking, connect with other community actors, and advocate for more inclusive food policymaking; and most importantly, leads to more impactful, fit-for-purpose, and equity-centred food policies that ensure everyone has the opportunity to achieve a healthy and culturally appropriate diet.

### Systemic gaps and tokenistic forms of community consultation in food policymaking

Arnstein’s Ladder of Citizen Participation [22] sought to describe the varied forms of community participation in decision-making. Many scholars have built on this work over the years and the terminology used to describe citizen participation and public engagement has evolved [32]. This framework can be applied to research, policy and practice – based on the extent to which participatory approaches allow community members to only voice their perspectives (i.e., more tokenistic forms) or support their power to influence the decisions that affect them [22]. Our study participants mentioned examples of higher degrees of community engagement outside the field of food policy, particularly in mental health. This highlights the opportunity to apply the same principles of effective community engagement observed in other health policy sectors to the field of food and nutrition. Notably, literature on community engagement in food and nutrition initiatives is often limited to varying forms of community consultation. For example, an analysis of food policy documents from high-income countries found ongoing gaps in acknowledging the need for community to be involved in all stages of policymaking [8]. In another study, US users of a food security program were invited to provide feedback on previously developed recommendations to advance equity in the program, with the aim to inform the renewal of the US Farm bill; however, users were not engaged in the latter part of the policymaking process [28]. Additionally, a large number of adolescents globally were engaged in participatory workshops to capture their lived experiences of food systems, including their perspectives on food sustainability and food security; once again, this engagement did not involve inclusive food policymaking [29]. Finally, Australian research echoing this notion found that Aboriginal organisations have attempted to influence food security and nutrition policies through formal policy consultations, with very few of their recommendations being adopted in final food policy documents [17, 33]. Collectively, this evidence suggests that even when governments, researchers and practitioners prioritise community consultations with people who experience marginalisation, systemic gaps persist in how decision-makers enable people with lived experience of marginalisation to co-lead and have shared ownership of population-level food and nutrition policy agendas and processes.

### Creating inclusive food policy processes

Recommended actions to achieve higher degrees of community engagement in food policymaking have been described in other studies and coincide with our participants’ reflections on what is required to achieve true collaboration. For example, adolescents from the previously mentioned global study highlighted the need for decision-makers (who are gatekeepers) to actively choose to work with them, listen to their lived experiences, and include them in food policymaking [34]. Another study describing recommendations for co-creating public health initiatives also emphasised the importance of effective recruitment strategies and commitment to representation [35]. Finally, several studies have described the importance of disseminating and using the outputs of engaging people with lived experience of marginalisation in equitable food policy advocacy [14, 36, 37]. We extend upon this previous evidence by discussing the importance of having a dedicated budget for conducting this type of work and doing no harm to the people engaged.

All the actions identified by participants in our study align with the principles and enablers for meaningful engagement of individuals living with non-communicable diseases, mental health conditions, and neurological conditions, as described in a recently published WHO framework [38]. For example, having a dedicated budget to ensure sufficient resources and adequate payments to the people engaged, is reflected by the WHO framework elements “sustainable financing” and “dignity and respect”. Our theme related to striving for true collaboration is reflected within the WHO’s enablers “institutionalising engagement”, “redistributing power” and “integrated approaches”, and speaks to the principles of “power and equity”, “inclusivity and intersectionality”, “commitment and transparency”, and “institutionalisation and contextualisation”. Our findings around doing no harm to the people engaged is reflected within the enabler “elimination of stigmatisation” and supports the principle “dignity and respect”. Finally, ensuring effective dissemination and advocacy is reflected within the enabler “capacity-building”, which includes creating the platforms for knowledge exchange [38]. The framework recommends tangible actions for the WHO and Member States to realise each of the enablers; for example, “remunerate individuals with lived experience for all engagements (100%) at a rate equivalent to that for technical experts” [38]. These recommendations echo what some of our participants have said and should be considered alongside our findings to support meaningful community engagement in food policy processes.

### Implications for policy and practice

The outcomes of meaningfully engaging people with lived experience of marginalisation in food policy, research, and practice, as discussed by our study participants, are similar to those that underpin traditional participatory action research. These include: (i) increasing decision-makers’ awareness of the social, structural and political determinants of health and how these disproportionally affect people experiencing marginalisation, (ii) increasing people’s confidence, self-value, sense of belonging, capacity, community networks, and amplifying people’s power to take action [39]; and (iii) more tailored and effective targeted and population policies [35, 40]. In recognition of these outcomes, there have been several calls to strengthen the engagement of people with lived experience of marginalisation in food policy, research, and practice [41–44]. Achieving this in the real-world may be limited by the slow nature of government processes, the high turnover within governmental institutions, and the lack of clear direction on how to effectively conduct this work specifically within the context of food policymaking. Nonetheless, we have developed a list of 10 practical recommendations for meaningfully engaging people with lived experience of marginalisation that researchers and decision-makers can implement to progress equity in food policymaking. The 10 recommendations stemming from our interpretation of participant interview data are:

1. Value people’s lived experience of marginalisation as an additional form of evidence to inform food policy processes and equity-oriented research.
2. Dedicate budget, sufficient time, and resources for meaningful engagement of people with lived experience of marginalisation.
3. Effectively recruit a diverse and representative group of people with lived experience of marginalisation into participatory food policy platforms.
4. Meet people experiencing marginalisation where they are (i.e., go to community) and facilitate their engagement using multiple and appropriate methodologies.
5. Move beyond community consultation, by creating partnerships, breaking down the siloed action areas, and working alongside people with lived experience of marginalisation throughout all stages of food policy development.
6. Equalise power imbalances. This will necessitate listening to people with lived experience of marginalisation and elevating their power to decide and act on food policy decisions that affect them.
7. Adequately reimburse participants for their time using flexible payment methods.
8. Put in place appropriate support structures to prevent and manage any risk of distress or re-traumatisation, thereby striving to ensure no harm is done to the people engaged.
9. Ensure food policy solutions are context and culture-specific and aligned with people’s priorities.
10. Use accessible communication and dissemination strategies to ensure the results from any engagement of people with lived experience of marginalisation are accessible. These results should be communicated to challenge any dominant power imbalances and advocate for equitable food policies.

### Strengths and limitations

This study is the first to collect in-depth qualitative data from diverse research, NGO, government, advocacy, and community representatives (with expertise in food or social policy and/or community engagement) to comprehensively summarise how engaging people with lived experience of various forms of marginalisation can be strengthened to create more effective and equitable food policies in Australia. Our diverse sample reflects a breadth of experiences and knowledge, yielding findings that are in line with the WHO framework for meaningful engagement of people living with non-communicable diseases, and mental health and neurological conditions [38]. Consequently, our findings are likely to be translatable to many other policy areas. Nevertheless, as with all qualitative research, it was not possible to capture the full spectrum of lived experiences of marginalisation. Though some participants had dual roles, combining lived experiences of marginalisation with their professional work, we acknowledge the need to validate our findings with a broader range of community members experiencing other forms of marginalisation as a next step.

Furthermore, the results are also constrained by the limited identification of tangible attempts to create inclusive food policy, and consequently, limited knowledge of specific equitable food and nutrition policy outcomes. Additional research and actions are needed to bridge this gap in empirical knowledge, further substantiating advocacy calls for increased prioritisation of inclusive food policy.

While our study also provides an in-depth exploration of the views of 24 participants, and every effort was made to garner a wide range of experiences in these views, the results cannot be considered to represent the totality of views on the topic. Participants were ultimately professionally employed, and future research may seek to validate and critique these findings considering people with alternate forms of marginalisation.

## Conclusions

This study explored participants’ knowledge of barriers and enablers for effective engagement of people with lived experience of marginalisation. We offer 10 key recommendations on the actions required to improve current practices, which may be used as a starting point by all actors seeking to achieve more inclusive, effective and equitable food policy processes that can help reduce inequities in healthy diets. At their core, these recommendations seek to challenge systems of discrimination by demonstrating how we can all tangibly shift to ways of working that value and elevate the power of people who are often excluded from many decision-making systems, specifically when it comes to food and nutrition.

## Electronic supplementary material

Below is the link to the electronic supplementary material.

Supplementary Material 1

## Data Availability

The datasets used and/or analysed during the current study are available from the corresponding author on reasonable request.

## Abbreviations

COREQ: COnsolidated criteria for REporting Qualitative research guidelines
LGBTIQA+: Lesbian, Gay, Bisexual, Transgender, Intersex, Queer/questioning, Asexual
NCDs: Non-Communicable diseases
NGOs: Non-Governmental organisations
UK: United Kingdom
VicHealth: Victorian health promotion foundation

## References

[1] Afshin A, Sur PJ, Fay KA, Cornaby L, Ferrara G, Salama JS, et al. Health effects of dietary risks in 195 countries, 1990–2017: a systematic analysis for the global burden of Disease Study 2017. Lancet. 2019;393(10184):1958–72. 30954305 10.1016/S0140-6736(19)30041-8 PMC6899507

[2] Bono F, Matranga D. Socioeconomic inequality in non-communicable diseases in Europe between 2004 and 2015: evidence from the SHARE survey. Eur J Pub Health. 2019;29(1):105–10. 30169634 10.1093/eurpub/cky165 PMC6345203

[3] Backholer K, Mannan HR, Magliano DJ, Walls HL, Stevenson C, Beauchamp A, et al. Projected socioeconomic disparities in the prevalence of obesity among Australian adults. Aust N Z J Public Health. 2012;36(6):557–63. 23216497 10.1111/j.1753-6405.2012.00885.x

[4] Zorbas CBJ, Chung A, Peeters A, Booth S, Pollard C, Allender S, Hawkes C, Isaacs A, Backholer K. Shifting the social determinants of food insecurity during the COVID-19 pandemic: the Australian experience. Food Secur. 2022. 10.1007/s12571-022-01318-4 PMC9483265 36160693

[5] Global action plan for the prevention. and control of noncommunicable diseases 2013–2020. World health Organization. Geneva, Switzerland. 2013.

[6] Action Agenda for Health. Promotion 2019–2023. Victorian Health Promotion Foundation (VicHealth).

[7] National Preventive Health. Strategy 2021–2030. Commonwealth of Australia. Department of Health; 2021.

[8] Zorbas C, Browne J, Chung A, Baker P, Palermo C, Reeve E et al. National nutrition policy in high-income countries: is health equity on the agenda? Nutrition Reviews. 2020. 10.1093/nutrit/nuaa120 33230539

[9] Kumanyika SK. A Framework for increasing equity impact in obesity Prevention. Am J Public Health. 2019;109(10):1350–7. 31415203 10.2105/AJPH.2019.305221 PMC6727309

[10] Friel S, Hattersley L, Ford L, O’Rourke K. Addressing inequities in healthy eating. Health Promot Int. 2015;30(suppl2):ii77–88. 26420812 10.1093/heapro/dav073

[11] Chung AZC, Peeters A, Backholer K, Browne J. A critical analysis of representations of inequalities in childhood obesity in Australian health policy documents. 2021 (forthcoming). 10.34172/ijhpm.2021.82 PMC9808209 34380204

[12] Browne J, Hayes R, Gleeson D. Aboriginal health policy: is nutrition the ‘gap’ in ‘Closing the gap’? Aust N Z J Public Health. 2014;38(4):362–9. 25091077 10.1111/1753-6405.12223

[13] Helson C, Walker R, Palermo C, Rounsefell K, Aron Y, MacDonald C, et al. Is Aboriginal nutrition a priority for local government? A policy analysis. Public Health Nutr. 2017;20(16):3019–28. 28803580 10.1017/S1368980017001902 PMC10261319

[14] Baker P, Friel S, Kay A, Baum F, Strazdins L, Mackean T. What enables and constrains the inclusion of the Social Determinants of Health Inequities in Government Policy agendas? A narrative review. Int J Health Policy Manage. 2018;7(2):101–11. 10.15171/ijhpm.2017.130 PMC5819370 29524934

[15] Nisbett N, Harris J, Backholer K, Baker P, Jernigan VBB, Friel S. Holding no-one back: the Nutrition Equity Framework in theory and practice. Global Food Secur. 2022;32:100605. 10.1016/j.gfs.2021.100605 PMC9983632 36873709

[16] Judelsohn A, Orom H, Leon D, Raja S. Refuge in new food environments? The role of urban planning in facilitating food equity for new americans. J Urban Affairs. 2021;43(6):872–89.

[17] Browne J, Gilmore M, Lock M, Backholer K. First Nations peoples’ participation in the Development of Population-wide food and Nutrition Policy in Australia: a Political Economy and Cultural Safety Analysis. Int J Health Policy Manage. 2020. 10.34172/ijhpm.2020.175 PMC9309971 33008258

[18] Browne J, Gleeson D, Adams K, Minniecon D, Hayes R. Strengthening Aboriginal and Torres Strait Islander health policy: lessons from a case study of food and nutrition. Public Health Nutr. 2019;22(15):2868–78. 31115277 10.1017/S1368980019001198 PMC10260607

[19] OECD. Eight Ways to Institutionalise Deliberative Democracy, OECD Public Governance Policy Paper. 2021. Available from: https://www.oecd-ilibrary.org/governance/eight-ways-to-institutionalise-deliberative-democracy_4fcf1da5-en

[20] Madison D. Critical ethnography: method, ethics and performance. United States of America: SAGE Publications, Inc; 2020.

[21] Sabatier P. Theories of the Policy Process, Second Edition (2nd ed.). Routledge. 2007 10.4324/9780367274689

[22] Arnstein SR. A ladder of Citizen Participation. J Am Inst Planners. 1969;35(4):216–24.

[23] Den Broeder L, Devilee J, Van Oers H, Schuit AJ, Wagemakers A. Citizen Science for public health. Health Promot Int. 2016;33(3):505–14. 10.1093/heapro/daw086 PMC6005099 28011657

[24] Suomi A, Freeman B, Banfield M. Framework for the engagement of people with a lived experience in program implementation and research. Review and report prepared for the LifeSpan suicide prevention project. Australian National University; 2020.

[25] Ayiwe E, Colom A, Cook A, Murray A, Parry LJ. Engaging people with lived experience: best practice, challenges, and opportunities. UK: The Democratic Society; 2022.

[26] Fisher J, Fones G, Arivalagan Y, Ahmadpour I, Akselrod S, Olsen M. WHO framework on meaningful engagement: a transformational approach to integrate lived experience in the noncommunicable disease and mental health agenda. PLOS Global Public Health. 2024;4(5):e0002312. 38809940 10.1371/journal.pgph.0002312 PMC11135697

[27] Tong A, Sainsbury P, Craig J. Consolidated criteria for reporting qualitative research (COREQ): a 32-item checklist for interviews and focus groups. Int J Qual Health Care. 2007;19(6):349–57. 17872937 10.1093/intqhc/mzm042

[28] Bronner SE. Of Critical Theory and Its Theorist. Second Edition. Routledge, New York and London. 2002.

[29] Graham ID, Logan J, Harrison MB, Straus SE, Tetroe J, Caswell W, et al. Lost in knowledge translation: time for a map? J Contin Educ Health Prof. 2006;26(1):13–24. 16557505 10.1002/chp.47

[30] Malterud K, Siersma VD, Guassora AD. Sample size in qualitative interview studies: guided by Information Power. Qual Health Res. 2016;26(13):1753–60. 26613970 10.1177/1049732315617444

[31] Knill C, Tosun J. Policy making. Konstanz, Germany: University of Konstanz; 2008.

[32] Slotterback CS, Lauria M. Building a Foundation for Public Engagement in Planning. J Am Plann Association. 2019;85(3):183–7.

[33] Christidis R, Lock M, Walker T, Egan M, Browne J. Concerns and priorities of Aboriginal and Torres Strait Islander peoples regarding food and nutrition: a systematic review of qualitative evidence. Int J Equity Health. 2021;20(1):220. 34620180 10.1186/s12939-021-01551-x PMC8499519

[34] Fleming CAK, Sharma D, Brunacci K, Chandra S, Lala G, Munn L, et al. Fix my food: an urgent call to action from adolescents on how they experience and want to see change in their food systems. J Hum Nutr Diet. 2023;36(6):2295–309. 37728211 10.1111/jhn.13228

[35] Leask CF, Sandlund M, Skelton DA, Altenburg TM, Cardon G, Chinapaw MJM, et al. Framework, principles and recommendations for utilising participatory methodologies in the co-creation and evaluation of public health interventions. Res Involv Engagem. 2019;5(1):1–16. 30652027 10.1186/s40900-018-0136-9 PMC6327557

[36] Izumi BT, Schulz AJ, Israel BA, Reyes AG, Martin J, Lichtenstein RL, et al. The one-pager: a practical policy advocacy tool for translating community-based participatory research into action. Prog Community Health Partnersh. 2010;4(2):141–7. 20543489 10.1353/cpr.0.0114 PMC2921614

[37] Restall G, Cooper JE, Kaufert JM. Pathways to translating experiential knowledge into mental health policy. Psychiatr Rehabil J. 2011;35(1):29–36. 21768075 10.2975/35.1.2011.29.36

[38] World Health Organization. WHO framework for meaningful engagement of people living with noncommunicable diseases, and mental health and neurological conditions. Geneva: World Health Organization; 2023.

[39] Akom A, Shah A, Nakai A, Cruz T. Youth Participatory Action Research (YPAR) 2.0: how technological innovation and digital organizing sparked a food revolution in East Oakland. Int J Qual Stud Educ. 2016;29(10):1287–307. 28835731 10.1080/09518398.2016.1201609 PMC5564687

[40] Browne J, Adams K, Atkinson P, Gleeson D, Hayes R. Food and nutrition programs for Aboriginal and Torres Strait Islander australians: an overview of systematic reviews. Aust Health Rev. 2018;42(6):689–97. 28923162 10.1071/AH17082

[41] Zorbas C, Jeyapalan D, Nunez V, Backholer K. Community lived experience should be central to food systems policy. Nat Food. 2023;4(1):7–9. 37118565 10.1038/s43016-022-00676-8

[42] Spires M, Battersby J, Cohen N, Daivadanam M, Demmler KM, Mattioni D, et al. The people’s Summit: a case for lived experience of food environments as a critical source of evidence to inform the follow-up to the 2021 UN Food Systems Summit. Global Food Secur. 2023;37:100690.

[43] Charlton J. Nothing About Us Without Us. Disability Oppression and Empowerment.2000.

[44] Hawkes C, Gallagher-Squires C, Spires M, Hawkins N, Neve K, Brock J, et al. The full picture of people’s realities must be considered to deliver better diets for all. Nat Food. 2024;5(11):894–900. 39420226 10.1038/s43016-024-01064-0

