# Supplementary material for: Equalising power imbalances or a trail of broken promises? A qualitative study on engaging people with diverse lived experience of marginalisation in food policymaking in Australia
Source: BMC Public Health. 2025 Feb 14;25:613. doi: 10.1186/s12889-025-21733-4 (PMC11827468; doi:10.1186/s12889-025-21733-4)
Supplement: Supplementary file 1 — Supplementary Material 1 [file 12889_2025_21733_MOESM1_ESM.docx]

**SUPPLEMENTARY MATERIAL:**

**Equalising power imbalances or a trail of broken promises? A qualitative study on engaging people with diverse lived experience in food policymaking**

Carolina Venegas Hargous^1^, Kevin Kapeke^2^, Kathryn Backholer^1^, Dheepa Jeyapalan^2^, Veronica Nunez^2^, Jennifer Browne^1^, Anna Peeters^1^, Alexandra Chung^3^, Steven Allender^1^, Victoria Stead^4^, Yin Paradies^5^, Christina Zorbas^1^

*^1^Global Centre for Preventive Health and Nutrition, School of Health and Social Development, Institute for Health Transformation, Faculty of Health, Deakin University, Geelong, Australia*

*^2^The Victorian Health Promotion Foundation (VicHealth), Melbourne, Australia*

*^3^Department of Nutrition, Dietetics and Food, Monash University, Melbourne, Australia*

*^4^School of Humanities and Social Sciences, Faculty of Arts and Education, Deakin University, Geelong, Australia*

*^5^Alfred Deakin Institute, Faculty of Arts and Education, Deakin University, Geelong, Australia*

**Table S1. Original semi-structured interview guide (not previously published) used to guide data collection based on key theoretical frameworks**

| **Part 1 introduction:** In this first part, we want to explore your thoughts on lived experiences of structural barriers to good health and nutrition. | |
| --- | --- |
| **Underpinning theory or evidence** | **Questions** |
| **Context**  **(critical theory, intersectionality theory, SDH*, and ACF^+^)** | 1. Based on what you know or have experienced, what are some of the things that make it hard to consume a healthy diet if you’re experiencing some form of structural disadvantage? 2. I am going to read you a quote from a single mother of three children who also works casually in Regional Victoria. In December 2020, she said to me that:   “***No one should be disadvantaged by the food that they have to eat… You have to choose between food or your house, or your warmth, or the internet for your children because they have to do schooling... We don't live in a third world country, but sometimes parts of it feel like it is.”***  What does this story of someone’s lived experience make you **think or feel**?  Prompts:   - Do you think the public understand what it’s like to struggle to access healthy foods? Why or why not? - Do you think our decision makers understand? Why or why not?  1. How might it be **relevant to you or your organisation** to better understand the intersection between people’s lived experiences of disadvantage and trying to access and consume healthy diets?   Prompts:   - What does the term ‘lived experience’ mean for you or your organisation? - What is the importance, if any, of someone’s ‘lived experience’ with structural barriers to good health and nutrition? |
| **Part 2 introduction:** Next, we want to explore your perspectives on including people’s experiences of structural barriers to good health and nutrition in policymaking processes related to food, including priority actions, potential challenges, enablers, and benefits. | |
| **Challenging the status quo (ACF)** | 1. In your opinion, are current food policy actions *(including things like restricting unhealthy food and drink marketing, taxing sugary drinks and less healthy options, mandating food labels, funding school lunches and food banks)* – across all levels of government – effective in meeting the real-world needs and experiences of people who experience structural barriers to good nutrition in Victoria/Australia/globally?   Prompts:   - **Can you please explain why or why not?** - Where are we falling short? - What are we doing well?  1. In your opinion, are community members that experience disadvantage adequately heard in policy making at the local, state and national levels when it comes to their health and nutrition needs?   Prompts:   - **Who is not being heard?** - **Who is being heard?** - **Is equity just about ticking boxes?**  1. What are your thoughts on the way people who experience structural barriers to good health and nutrition are represented by policymakers across levels of government?   Prompts:   - Do we need to change these representations? Why or why not? **What can we do?** - **Are issues of intersectionality being adequately addressed?** Why or why not?  1. What are the major challenges, if any, to including the voices of these community members in food or health policymaking processes?   Prompts:   - Lack of evidence on how to act on the upstream determinants of health inequities (i.e., the evidence/policy gap) - No guidance on how, and no platforms available, to work with community members - No direct communication with government - Lack of government or high-profile champions  1. Can you think of any reasons why now might be a good time to include the voices of people who experience structural barriers to health in food policymaking processes?   Prompts:   - COVID-19 and other global events - Economic crises - Educational inequalities - Digital technology  1. What are the potential benefits, if any, of including these voices in food policymaking processes?   Prompts:   - Community empowerment - New policy learnings - Greater impact and action – reduce health inequities - For your organisation? - For the government? |
| **Part 3 introduction:** Thank you for your responses so far. We’re now at the last section. My final questions are about the actions you think should be prioritised to help develop policy processes that better include the voices of people who experience structural barriers to good nutrition. | |
| **Priority actions (ACF, existing evidence)** | 1. Whose responsibility is it to drive advocacy efforts and policy change to reduce inequalities in nutrition? Could you please elaborate on why you think this?   Prompts:   - What is my role as a researcher? - What is your role and that of your organisation? - What role should community members play?  1. Can you describe any examples of where people’s lived experience has or is being incorporated in efforts to drive policy action/s? This may or may not be in relation to nutrition or public health more broadly.   Prompts:   - Do you think it has been effective? Why or why not?  1. How do you think we can effectively communicate people’s everyday experiences with disadvantage and food insecurity to the public and policymakers to influence policy?   Prompts:   - What comms tools or outputs would you find most useful? How would you use these resources? - What can we learn from communications and media experts? - Do you think that language, narratives, and communication matter in driving change? Why our why not?  1. If you were to prioritise next steps for developing food policy processes that include the voices of people experiencing the most structural barriers to good nutrition, what would they be?   Prompts:   - If you had one or two messages for the Prime Minister or Ministers that are in positions to support policies to reduce inequalities in nutrition, what would they be? |

*Social Determinants of Health, ^+^Advocacy Coalition Framework

**Table S2. Participant characteristics**

| **Sector** | **Participant number** | **Expertise** |
| --- | --- | --- |
| *Research (n=5)* | *Participant 4* | *Researcher focused on equitable food policies in Australia* |
|  | *Participant 14* | *Researcher focused on equitable food policies in Australia* |
|  | *Participant 7* | *Researcher focused on policies to improve First Nations nutrition* |
|  | *Participant 1* | *Researcher focused on equitable food policies in the UK* |
|  | *Participant 13* | *Researcher focused on equitable food policies in Africa* |
| *NGOs, advocates & community representatives (n=9)* | *Participant 12* | *Food policy advocate employed by an NGO in Victoria* |
|  | *Participant 8* | *Policy advocate employed in the social services sector to work with people who experience marginalisation in Western Australia* |
|  | *Participant 9* | *Communications officer with experience working with people with lived experience of marginalisation in mental health settings* |
|  | *Participant 11* | *Youth wellbeing advocate in regional Victoria* |
|  | *Participant 2* | *Public health advocate employed by an NGO in Victoria* |
|  | *Participant 6* | *Nutritionists with experience working with First Nations communities in Australia* |
|  | *Participant 10* | *Expertise in facilitating lived experience engagement across health and social sectors in Australia* |
|  | *Participant 5* | *Lived experience representative from a community advisory group in Western Australia* |
|  | *Participant 15* | *LGBTIQ+ youth wellbeing advocate in Victoria* |
| *Government (n=10)* | *Participant 16* | *Public health unit officer in Victoria with a migrant background* |
|  | *Participant 17* | *Victorian State government representative with expertise in health and social policy* |
|  | *Participant 3* | *Local government health/nutrition officer in Victoria* |
|  | *Participant 18* | *Local government health/nutrition officer in Victoria* |
|  | *Participant 19* | *Local government health/nutrition officer in Victoria* |
|  | *Participant 20* | *Local government health/nutrition officer in Victoria* |
|  | *Participant 21* | *Local government health/nutrition officer in Victoria* |
|  | *Participant 22* | *Local government health/nutrition officer in Victoria* |
|  | *Participant 23* | *Local government health/nutrition officer in Victoria* |
|  | *Participant 24* | *Local government health/nutrition officer in Victoria* |
